# Effect of Experiment Warming on Soil Fungi Community of *Medicago sativa*, *Elymus nutans* and *Hordeum vulgare* in Tibet

**DOI:** 10.3390/jof9090885

**Published:** 2023-08-29

**Authors:** Zhiming Zhong, Guangyu Zhang, Gang Fu

**Affiliations:** Lhasa Plateau Ecosystem Research Station, Key Laboratory of Ecosystem Network Observation and Modeling, Institute of Geographic Sciences and Natural Resources Research, Chinese Academy of Sciences, Beijing 100101, China

**Keywords:** soil microbiological diversity, cooccurrence network, α-diversity, temperature sensitivity

## Abstract

The uncertainty response of soil fungi community to climate warming in alpine agroecosystems will limit our ability to fully exploit and utilize soil fungi resources, especially in alpine regions. In this study, a warming experiment was conducted in one perennial leguminous agroecosystem [i.e., alfalfa (*Medicago sativa*)], perennial gramineous agroecosystem (i.e., *Elymus nutans*) and annual gramineous agroecosystem [i.e., highland barley (*Hordeum vulgare* L)] in Tibet since 2016 to investigate the response of soil fungi community to climate warming. Soils at two layers (i.e., 0–10 cm and 10–20 cm) were collected in August 2017 to estimate soil fungi community based on the ITS method. The α-diversity, community composition and functional group abundance of soil fungi in the leguminous agroecosystem were more sensitive to climate warming. The α-diversity of soil fungi in the perennial gramineous agroecosystem were more sensitive to climate warming, but topology parameters of soil fungi species cooccurrence network in the annual gramineous agroecosystem were more sensitive to climate warming. Compared with 0–10 cm, soil fungal α-diversity, community composition and functional group abundance at 10–20 cm were more sensitive to climate warming. The topological parameters of soil fungi species cooccurrence network at 0–10 cm in the gramineous agroecosystem were more sensitive to climate warming, but those at 10–20 cm in the leguminous agroecosystem were more sensitive to climate warming. Warming increased the differences of soil fungi α-diversity and functional composition. For the *Medicago sativa* agroecosystem, warming increased the abundance of soil pathogenic fungi but decreased the abundance of soil symbiotic and saprophytic fungi at 10–20 cm. Therefore, responses of the soil fungi community to climate warming varied with agroecosystem types and soil depth. Climate warming can alter the differences of the soil fungi community among agroecosystems. Changes in soil fungi community caused by climate warming may be detrimental to the growth of alpine crops, at least for perennial *Medicago sativa* in Tibet.

## 1. Introduction

The number of people on Earth is increasing rapidly and is expected to exceed 9 billion by 2050 [1]. Feeding a growing population is one of the biggest challenges in global food production. To solve this problem, global food production needs to increase by 70% to meet demands by 2050 [2]. With the continuous improvement of human living standards, human needs for livestock products (such as milk, meat, etc.) are also increasing. As the main battleground for grazing, natural grasslands may not only suffer from probable increasing grazing pressure (e.g., large loss of forage stock) but also from the probable adverse effects of climate change (e.g., the decline in forage nutrition quality and temporal stability of plant production caused by climate change) [3]. All these put forward new requirements for the food production capacity of agroecosystems and the high-quality development of animal husbandry. To ease the pressure on natural grasslands for grazing and the increasing demand for livestock products from a growing population, artificial forage cultivation is gaining acceptance in recent years. Both food production and artificial forage production depend mainly on farmland soils, and soil quality is a big determinant of food production and artificial forage production [4]. As an important part of a soil system, cultivated soil fungi are not only the core of restoring soil quality of degraded cultivated land but also the core of maintaining, stabilizing and improving the quality of non-degraded cultivated land [4,5]. Under the background of global warming, soil fungi in farmlands are inevitably affected by climate warming. Therefore, understanding the influences of climate warming on soil fungi community in agroecosystems is an important theoretical basis for fully exploiting beneficial soil fungi resources to serve global food security and animal product security.

Alpine agroecosystems on the Qinghai–Tibet Plateau are not only important parts of global alpine agroecosystems but also the main battlefield of highland barley production and artificial forage production on the Plateau [4]. Improving soil quality of alpine agroecosystems is an important guarantee to improve the yield of barley and forage grass, and soil fungi community may be the core of soil quality improvement [6]. In order to explore the responses and related driving mechanisms of these alpine agroecosystems to climate warming, more and more warming experiments have been carried out in alpine agroecosystems on the Qinghai–Tibet Plateau in recent years, although related studies are still relatively less (<30). Moreover, previous studies have mainly investigated the effects of warming on soil respiration in highland barley and the growth of highland barley [7], wheat growth [8] and oat growth [9], and only a few studies have tried to examine the effects of warming on soil fungi community in the highland barley agroecosystem [4] on the Tibetan Plateau. No studies have investigated the response of leguminous agroecosystems to warming on the Tibetan Plateau. No studies have tried to investigate the effect of warming on the species cooccurrence network and community assembly mechanisms of soil fungi community in agroecosystems on the Tibetan Plateau. The soil fungi species cooccurrence network can reflect complex relationships among soil fungi species, but soil fungal community assembly mechanisms can quantify the relative effects of selection and dispersal processes on soil fungal community. These uncertainties limit our understanding of the changes of soil fungi community under the background of climate warming, thus limiting the mining and efficient using of beneficial soil fungi resources in alpine agroecosystems on the Tibetan Plateau. Therefore, it is necessary to strengthen the research on the response of soil fungi community to warming in alpine agroecosystems.

In this study, a warming experiment was conducted in three agroecosystems (i.e., *Medicago sativa*, *Elymus nutans* and *Hordeum vulgare*) of Tibet since April 2016. *Hordeum vulgare* is one of the main crops in Tibet. *Elymus nutans* is not only a wild gramineous forage commonly found in natural grasslands but also one of the most common cultivated forages in Tibet. Compared to gramineous forages, *Medicago sativa* often has a higher crude protein content, so it is favored by the livestock breeding industry in Tibet. Moreover, *Medicago sativa* can adapt to harsh environmental conditions (e.g., low temperature) in Tibet. Therefore, this study chose the three agroecosystems as the study objects. The main objectives were to compare the different effects of warming on soil fungi community (e.g., total abundance, α-diversity, community composition, community assembly, species cooccurrence network) (1) between leguminous and gramineous crops agroecosystems and (2) between perennial and annual gramineous crops agroecosystems of Tibet. This study can provide some services for food security, highland barley security, high-quality development of animal husbandry, rural revitalization, etc., on the Qinghai–Tibet Plateau, at least for Tibet.

## 2. Materials and Methods

### 2.1. Study Area and Experiment Design

The warming experiment included 6 treatments with 5 replicates, and, in total, there were 30 experimental plots. All the 30 experiment plots were set up in the Lhasa Agroecosystem Research Station (91°21′ E, 29°41′ N, 3688 m) on 15 April 2016. There were two experiment factors (warming × agroecosystem). Open top chambers (2 m × 2 m × 2 m) were used to elevate temperature throughout the year since 15 April 2016. There were three agroecosystems, including the *Medicago sativa*, *Elymus nutans* and *Hordeum vulgare* agroecosystems. *Medicago sativa* belongs to leguminous crops, but both *Elymus nutans* and *Hordeum vulgare* belong to gramineous crops. Both *Medicago sativa* and *Elymus nutans* are perennial crops, but *Hordeum vulgare* is an annual crop. All the three plant species were grown on 15 April 2016. *Hordeum vulgare* was harvested on 19 August 2016. *Hordeum vulgare* was grown on April 16 and harvested on 18 August 2017. Mean annual precipitation and mean annual air temperature was about 425 mm and 7.9 °C, respectively [4,7]. The HOBO microclimate stations (Onset Computer, Bourne, MA, USA) were used to monitor soil temperature (*T*_s_, 0.05 m) in 2016–2017. The HOBO microclimate stations have been widely used in the field warming experiment, and they work well in Tibet. Open top chambers increased *T*_s_ by 1.13 °C by 18 August 2017, which was comparable to the warming magnitude reported by a recent study [4].

### 2.2. Soil Sampling and Analysis

We collected soil samples at two layers (i.e., 0–10 cm and 10–20 cm) in August 2017. Within each quadrate (2 m × 2 m), a soil drill with a diameter of 4 cm was used to randomly collect soil subsamples, and this was treated as the soil sample of this quadrate after mixing. An ultra-low temperature freezer (–80 °C) was used to store all the soil samples before extraction of soil DNA. The amplify barcoded primers of soil fungi were ITS1F: 5′-CTTGGTCATTTAGAGGAAGTAA-3′; ITS2R: 5′-GCTGCGTTCTTCATCGATGC-3′ [4]. The PCR amplification processes included a pre-degeneration process (i.e., the reaction was at 98 °C for 1 min), 27 cycling process (i.e., at 98 °C for 30 s, 55 °C for 30 s and 72 °C for 30 s) and an extension process (i.e., the reaction was continued at 72 °C for 5 min) [4]. All the PCR amplification processes were performed in a 20 μL reaction system [4]. The library was sequenced using an Illumina Hiseq 2500 platform (250 bp paired-end reads). Reads, containing >10% of unknown nucleotides and <80% of bases with Q-value > 20, were removed [4]. The filtered reads were assembled into labels based on overlaps of paired-end reads whose overlap was >10 bp and mismatch was <2%. We obtained the operational taxonomic unit (OTU) using the Mothur software. We identified soil fungal species (i.e., OTU) using the UNITE database [4].

### 2.3. Statistical Analyses

We obtained taxonomic α-diversity (OTU number, Chao1_t_, ACE_t_, Shannon_t_, Simpson_t_) using the microtable$cal_alphadiv function of the microeco package. We obtained soil fungi functional data using the trans_func class of the microeco package. Only ‘Highly Probable’ and ‘Probable’ confidence ranking levels of soil fungi functional data were used. Then, we also obtained functional α-diversity (function number, Chao1_f_, ACE_f_, Shannon_f_, Simpson_f_) using the microtable$cal_alphadiv function of the microeco package. We obtained soil fungi functional abundance of the seven trophic groups (i.e., symbiotroph, pathotroph, saprotroph, symbiotroph–pathotroph, symbiotroph–saprotroph, pathotroph–saprotroph, symbiotroph–pathotroph–saprotroph). We obtained phylogenetic α-diversity (PD: Faith’s phylogenetic diversity; MPD: mean pairwise distance; MNTD: mean nearest taxon distance) using the pd, mpd and mntd functions of the picante package, respectively. We obtained the phylogenetic β-diversity matrix (βMNTD), which can reflect phylogenetic composition, using the bmntd.big function of the iCAMP package. We obtained the ecological process (i.e., heterogeneous selection, homogeneous selection, dispersal limitation, homogenizing dispersal, drift and others) of soil fungi community assembly using the icamp.big function of the iCAMP package [10]. We obtained the cooccurrence network topology parameter (i.e., vertex, edge, average degree, average path length, network diameter, clustering coefficient, density, heterogeneity, centralization) using the packages of microeco and meconetcomp. We also obtained the roles of all vertices in the cooccurrence network and plotted them using the packages of microeco and meconetcomp. We obtained the shared and exclusive species, vertex and edge of the cooccurrence network among the six treatments using the trans_venn function of the microeco package. We obtained the significant differences of species among the six treatments using the trans_diff function (i.e., linear discriminant analysis, LEfSe method) of the microeco package.

We obtained the main and interactive effects of warming and agroecosystem on α-diversity (i.e., taxonomic, functional and phylogenetic α-diversity), soil fungi function trophic abundance, ecological process of community assembly and topology parameter of the cooccurrence network using two-way variance analysis (i.e., stats package). We obtained the main and interactive effects of warming and agroecosystem on community composition (i.e., taxonomic, functional and phylogenetic composition) using two-way adonis2 (i.e., vegan package). We obtained the effect of warming on community composition for each one of the three agroecosystems using one-way adonis2. We obtained the effect of agroecosystem on community composition under control and warming conditions using one-way adonis2. We obtained the plots of the warming effects on α-diversity, soil fungi function trophic abundance, ecological process of community assembly and topology parameter of the cooccurrence network using the ggbarplot, stat_compare_means and ggarrange functions of the ggpubr package. We obtained the differences of α-diversity, soil fungi function trophic abundance, ecological process of community assembly and topology parameter of the cooccurrence network among the three agroecosystems under control and warming conditions using one-way variance analysis and Duncan multiple comparison. All the statistical analyses were conducted on the R4.2.2.

## 3. Results

### 3.1. Total Abundance

**Figure 1:**
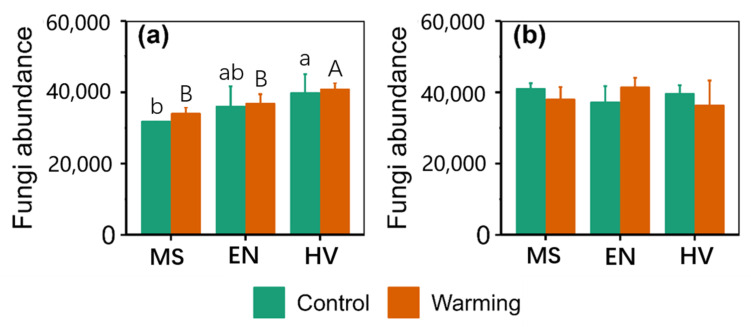
Comparison of soil fungi abundance between the control and warming conditions at (**a**) 0–10 cm and (**b**) 10–20 cm. MS: *Medicago sativa*; EN: *Elymus nutans*; HV: *Hordeum vulgare*. The lowercase letters indicate significant differences among *Medicago sativa*, *Elymus nutans* and *Hordeum vulgare* under control treatment. The uppercase letters indicate significant differences among *Medicago sativa*, *Elymus nutans* and *Hordeum vulgare* under warming treatment. No letters indicates non-significant differences among *Medicago sativa*, *Elymus nutans* and *Hordeum vulgare*.

There were significant main effects of agroecosystem on total abundance at 0–10 cm (Table S1). Total abundance of the *Hordeum vulgare* agroecosystem was 25.38% and 20.10% greater than that of the *Medicago sativa* agroecosystem under the control and warming conditions at 0–10 cm, respectively (Figure 1). Total abundance of the *Hordeum vulgare* agroecosystem was 10.81% greater than that of the *Elymus nutans* agroecosystem under warming conditions at 0–10 cm (Figure 1).

### 3.2. α-Diversity

**Figure 2:**
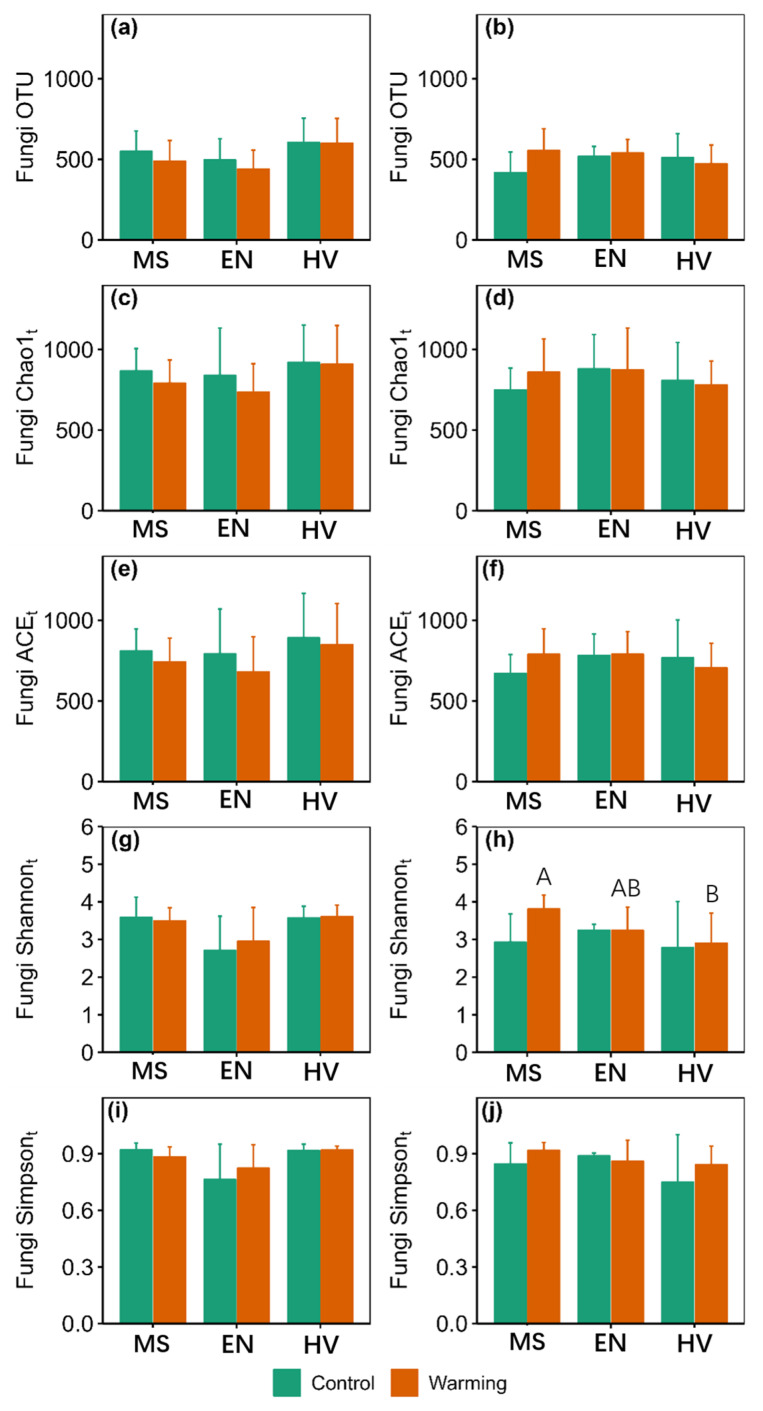
Comparison of soil fungi taxonomic α-diversity between the control and warming conditions at 0–10 cm (**a**,**c**,**e**,**g**,**i**) and 10–20 cm (**b**,**d**,**f**,**h**,**j**). MS: *Medicago sativa*; EN: *Elymus nutans*; HV: *Hordeum vulgare*. The uppercase letters indicate significant differences among *Medicago sativa*, *Elymus nutans* and *Hordeum vulgare* under warming treatment. No letters indicates non-significant differences among *Medicago sativa*, *Elymus nutans* and *Hordeum vulgare*.

**Figure 3:**
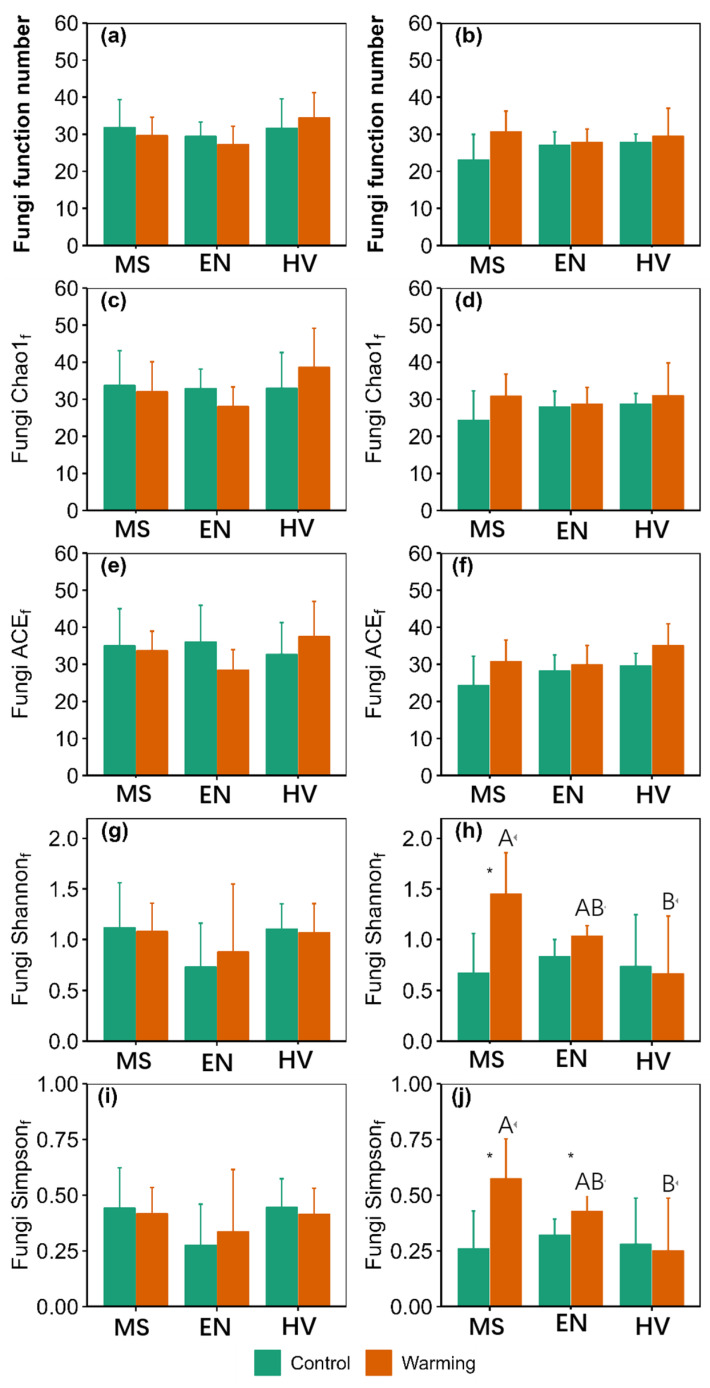
Comparison of soil fungi function α-diversity between the control and warming conditions at 0–10 cm (**a**,**c**,**e**,**g**,**i**) and 10–20 cm (**b**,**d**,**f**,**h**,**j**). MS: *Medicago sativa*; EN: *Elymus nutans*; HV: *Hordeum vulgare*. * indicate significant difference between the control and warming treatments for each plant species at *p* < 0.05, whereas no * indicates non-significant difference. The uppercase letters indicate significant differences among *Medicago sativa*, *Elymus nutans* and *Hordeum vulgare* under warming treatment. No letters indicates non-significant differences among *Medicago sativa*, *Elymus nutans* and *Hordeum vulgare*.

**Figure 4:**
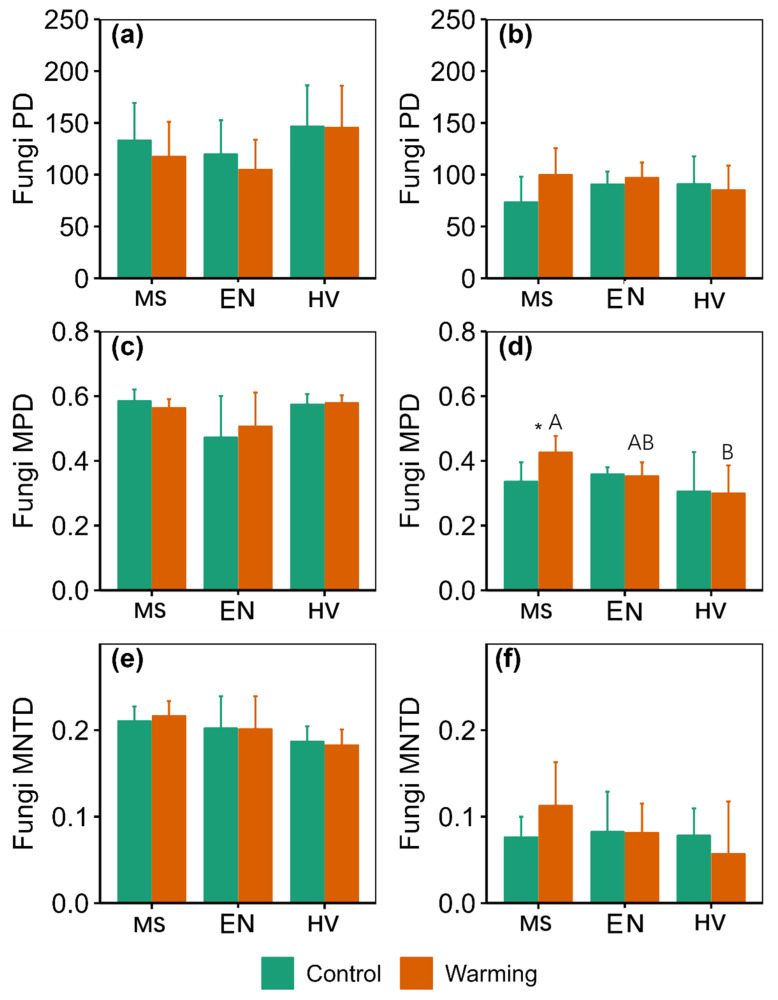
Comparison of soil fungi phylogenetic α-diversity between the control and warming conditions at 0–10 cm (**a**,**c**,**e**) and 10–20 cm (**b**,**d**,**f**). MS: *Medicago sativa*; EN: *Elymus nutans*; HV: *Hordeum vulgare*. * indicate significant difference between the control and warming treatments for each plant species at *p* < 0.05, whereas no * indicates non-significant difference. The uppercase letters indicate significant differences among *Medicago sativa*, *Elymus nutans* and *Hordeum vulgare* under warming treatment. No letters indicates non-significant differences among *Medicago sativa*, *Elymus nutans* and *Hordeum vulgare*.

There were significant main effects of warming on ACE_f_, Shannon_f_ and Simpson_f_ at 10–20 cm (Table S2). There were significant main effects of agroecosystem on Shannon_t_, Simpson_t_ and MPD at 0–10 cm (Table S2). Warming did not affect taxonomy α-diversity (Figure 2). Warming increased Simpson_f_ by 33.23% in the *Elymus nutans* agroecosystem and Shannon_f_, Simpson_f_ and MPD by 116.34%, 122.04% and 26.88% in the *Medicago sativa* agroecosystem at 10–20 cm, respectively (Figure 3 and Figure 4).

Shannon_t_, MPD, Shannon_f_ and Simpson_f_ in the *Hordeum vulgare* agroecosystem were 23.89%, 29.72%, 54.10% and 56.33% lower than those of the *Medicago sativa* agroecosystem at 10–20 cm under warming conditions, respectively (Figure 2, Figure 3 and Figure 4).

### 3.3. Community Composition

There was a significant main effect of warming on function composition at 10–20 cm (Table S3). Warming altered taxonomy composition and function composition in the *Medicago sativa* agroecosystem at 10–20 cm (Table S4). There were significant differences of function composition among the three agroecosystems at 10–20 cm under warming conditions (Table S5).

There were 374 and 338 common species among the 6 treatments at 0–10 and 10–20 cm, respectively (Figures S1 and S2). There were some exclusive species for any one of these six treatments (Figures S1 and S2). There were 20 and 23 taxa showing significant differences among the 6 treatments at 0–10 cm and 10–20 cm, respectively (Figures S3 and S4).

**Figure 5:**
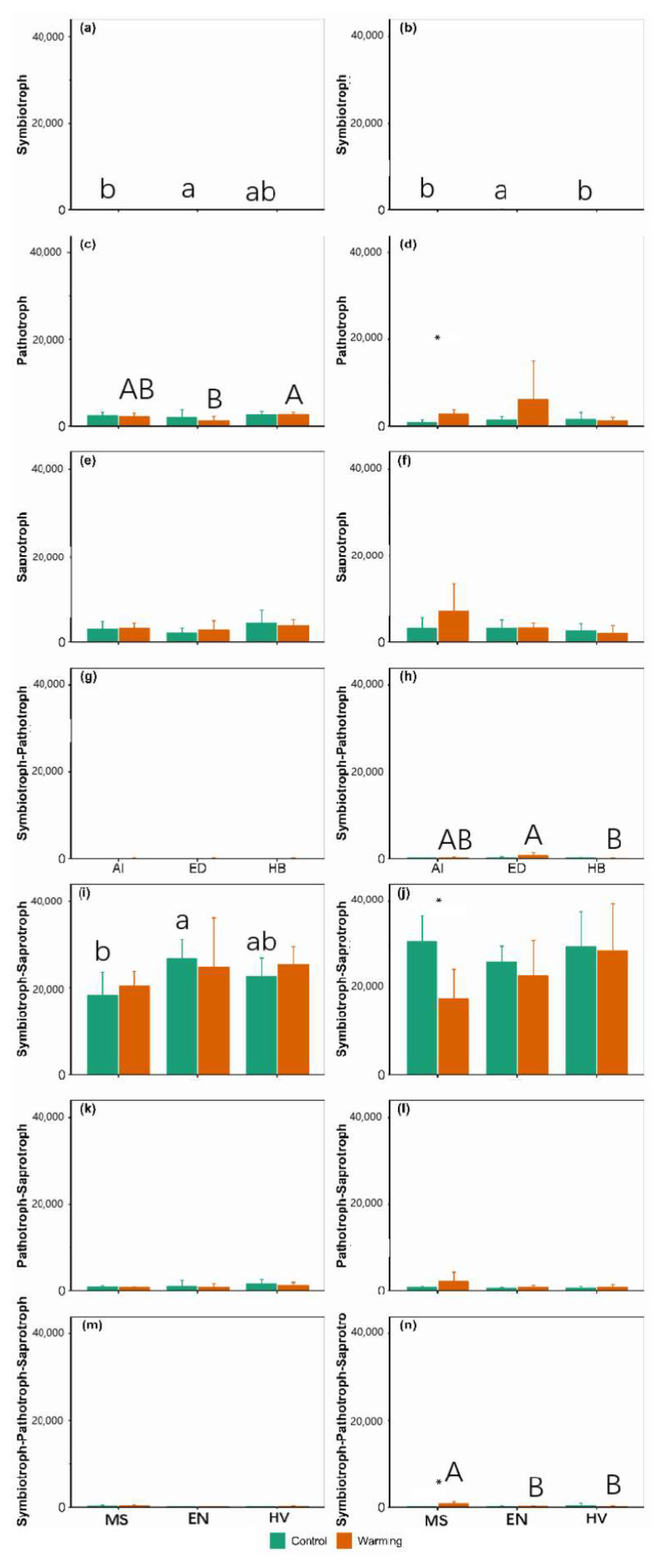
Comparison of ecological function of the soil fungi community between the control and warming conditions at 0–10 cm (**a**,**c**,**e**,**g**,**i**,**k**,**m**) and 10–20 cm (**b**,**d**,**f**,**h**,**j**,**l**,**n**). MS: *Medicago sativa*; EN: *Elymus nutans*; HV: *Hordeum vulgare*. * indicate significant difference between the control and warming treatments for each plant species at *p* < 0.05, whereas no * indicates non-significant difference. The lowercase letters indicate significant differences among *Medicago sativa*, *Elymus nutans* and *Hordeum vulgare* under control treatment. The uppercase letters indicate significant differences among *Medicago sativa*, *Elymus nutans* and *Hordeum vulgare* under warming treatment. No letters indicates non-significant differences among *Medicago sativa*, *Elymus nutans* and *Hordeum vulgare*.

There were significant main effects of warming on symbiotroph and symbiotroph–saprotroph fungi abundance at 10–20 cm and symbiotroph–pathotroph fungi abundance at 0–10 cm (Table S6). There were significant main effects of agroecosystem on symbiotroph and symbiotroph–pathotroph fungi abundance at 10–20 cm (Table S6). There were significant interactive effects of warming and agroecosystem on symbiotroph and symbiotroph–pathotroph–saprotroph fungi abundance at 10–20 cm (Table S6). Among the seven fungi function groups, the symbiotroph–saprotroph fungi abundance was the largest (Figure 5). Warming increased pathotroph fungi by 213.63% (i.e., 1827.00) and symbiotroph–pathotroph–saprotroph fungi by 527.86% (i.e., 674.6) but decreased symbiotroph–saprotroph fungi by 42.77% (i.e., 13091) in the *Medicago sativa* agroecosystem at 10–20 cm (Figure 5). Symbiotroph and symbiotroph–saprotroph fungi in the *Elymus nutans* agroecosystem were 269.86% and 45.93% greater than those of the *Medicago sativa* agroecosystem at 0–10 cm under control conditions, respectively (Figure 5). Pathotroph fungi in the *Hordeum vulgare* agroecosystem were 113.00% greater than those of the *Elymus nutans* agroecosystem at 0–10 cm under warming conditions (Figure 5). Symbiotroph fungi in the *Medicago sativa* and *Hordeum vulgare* agroecosystems were 89.35% and 98.67% lower than those of the *Elymus nutans* agroecosystem at 10–20 cm under control conditions, respectively (Figure 5). Symbiotroph–pathotroph fungi in the *Hordeum vulgare* agroecosystem were 87.03% lower than those of the *Elymus nutans* agroecosystem at 10–20 cm under warming conditions (Figure 5). Symbiotroph–pathotroph–saprotroph fungi in the *Elymus nutans* and *Hordeum vulgare* agroecosystems were 71.96% and 81.51% lower than those of the *Medicago sativa* agroecosystem at 10–20 cm under warming conditions, respectively (Figure 5).

### 3.4. Community Assembly

There were significant main effects of warming on homogeneous selection and drift and others process at 0–10 cm (Table S7). There were significant main effects of agroecosystem on dispersal limitation and drift and others process at 0–10 cm and homogeneous selection process at 10–20 cm (Table S7). There were significant interactive effects of warming and agroecosystem on heterogeneous selection, homogeneous selection and dispersal limitation process at 10–20 cm (Table S7).

**Figure 6:**
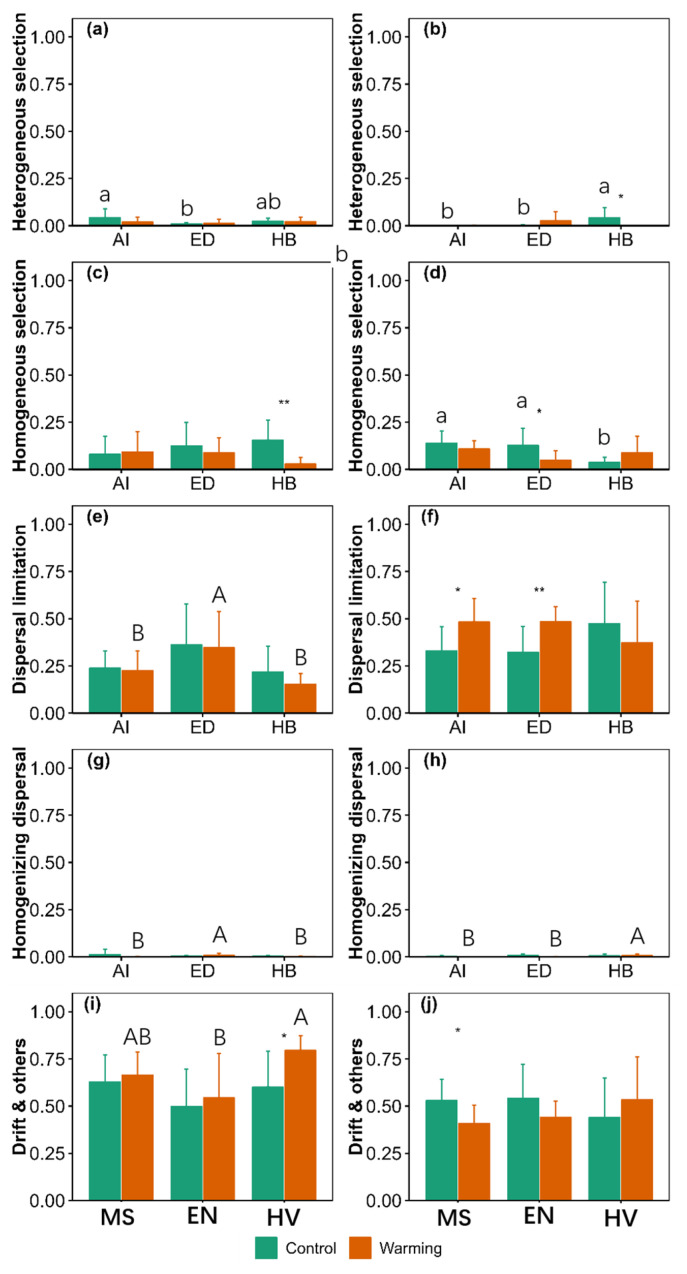
Comparison of ecological process of soil fungi community assembly between the control and warming conditions at 0–10 cm (**a**,**c**,**e**,**g**,**i**) and 10–20 cm (**b**,**d**,**f**,**h**,**j**). MS: *Medicago sativa*; EN: *Elymus nutans*; HV: *Hordeum vulgare*. * and ** indicate significant difference between the control and warming treatments for each plant species at *p* < 0.05 and *p* < 0.01, respectively, whereas no * or ** indicates non-significant difference. The lowercase letters indicate significant differences among *Medicago sativa*, *Elymus nutans* and *Hordeum vulgare* under control treatment. The uppercase letters indicate significant differences among *Medicago sativa*, *Elymus nutans* and *Hordeum vulgare* under warming treatment. No letters indicates non-significant differences among *Medicago sativa*, *Elymus nutans* and *Hordeum vulgare*.

For the *Medicago sativa* agroecosystem, warming increased dispersal limitation process by 46.55% but decreased drift and others process by 22.99% at 10–20 cm (Figure 6). For the *Elymus nutans* agroecosystem, warming increased dispersal limitation process by 50.41% but decreased homogeneous selection process by 62.22% at 10–20 cm (Figure 6). For the *Hordeum vulgare* agroecosystem, warming increased drift and others process by 32.43% at 0–10 cm but decreased homogeneous selection process by 81.51% at 0–10 cm and heterogeneous selection process by 98.12% at 10–20 cm (Figure 6).

Heterogeneous selection in the *Medicago sativa* agroecosystem was 324.01% greater than that of the *Elymus nutans* agroecosystem at 0–10 cm under control conditions (Figure 6). Dispersal limitation and homogenizing dispersal in the *Medicago sativa* and *Hordeum vulgare* agroecosystems were 35.51% and 91.05% and 56.03% and 80.21% lower than those of the *Elymus nutans* agroecosystem at 0–10 cm under warming conditions, respectively (Figure 6). Drift and others in the *Hordeum vulgare* agroecosystem was 46.33% greater than that of the *Elymus nutans* agroecosystem at 0–10 cm under warming conditions (Figure 6). Heterogeneous selection in the *Medicago sativa* and *Elymus nutans* agroecosystems was 97.81% and 94.38% lower than that of the *Hordeum vulgare* agroecosystem at 10–20 cm under control conditions (Figure 6). Homogeneous selection in the *Medicago sativa* and *Elymus nutans* agroecosystems was 267.85% and 238.12% greater than that of the *Hordeum vulgare* agroecosystem at 10–20 cm under control conditions (Figure 6). Homogenizing dispersal in the *Medicago sativa* and *Elymus nutans* agroecosystems was 92.23% and 88.65% lower than that of the *Hordeum vulgare* agroecosystem at 10–20 cm under warming conditions, respectively (Figure 6).

### 3.5. Cooccurrence Network

The proportion of positive edges to total edges was 96.05–99.83% and 96.33–99.79% at 0–10 and 10–20 cm, respectively (Figures S5 and S6). There were significant main effects of warming on edge, average degree, density, heterogeneity and centralization at 0–10 cm and clustering coefficient at 10–20 cm (Table S8). There were significant main effects of agroecosystem on average degree, heterogeneity and centralization at 0–10 cm and density and centralization at 10–20 cm (Table S8). There were significant interactive effects of warming and agroecosystem on average degree, density, heterogeneity and centralization at 10–20 cm (Table S8). There were shared vertices among the six treatments (Figures S7 and S8).

**Figure 7:**
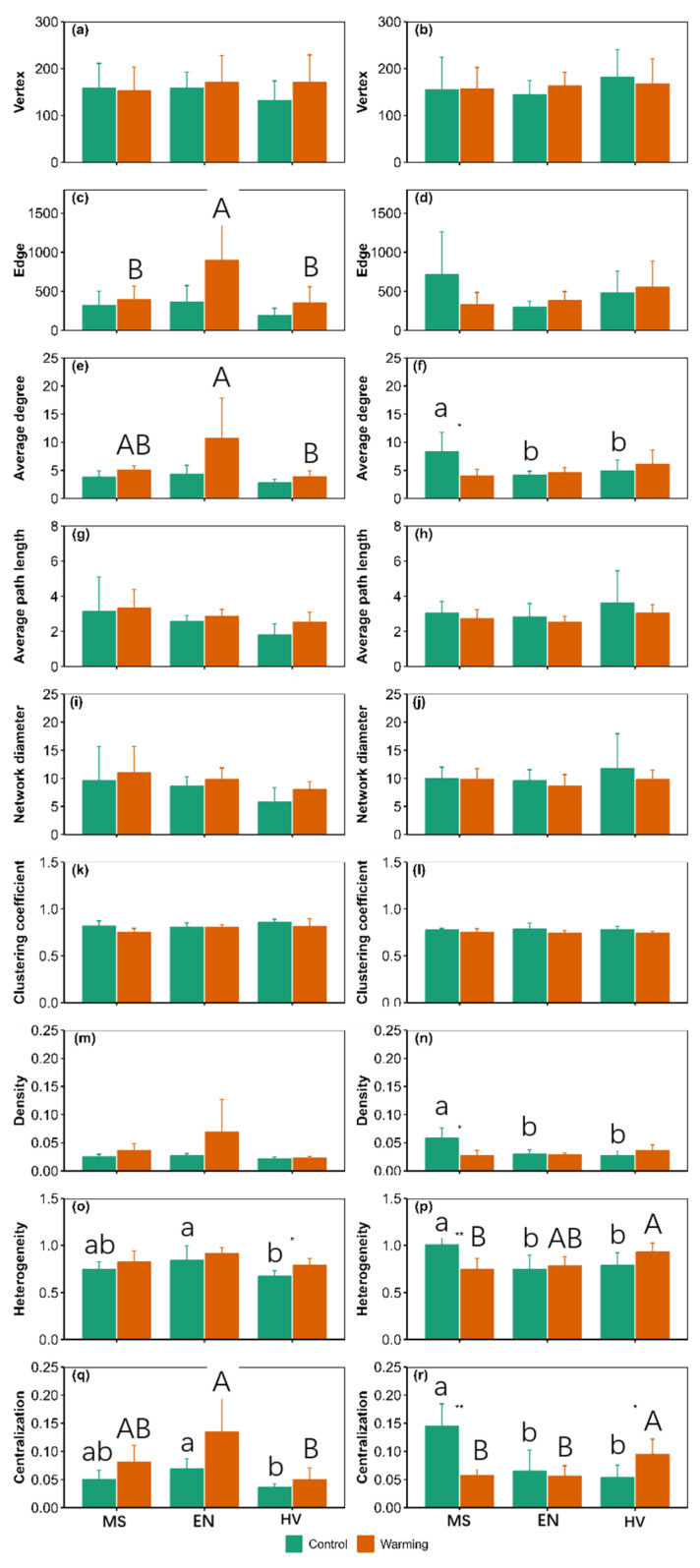
Comparison of the cooccurrence network topology parameter of soil fungi community between the control and warming conditions at 0–10 cm (**a**,**c**,**e**,**g**,**i**,**k**,**m**,**o**,**q**) and 10–20 cm (**b**,**d**,**f**,**h**,**j**,**l**,**n**,**p**,**r**). MS: *Medicago sativa*; EN: *Elymus nutans*; HV: *Hordeum vulgare*. * and ** indicate significant difference between the control and warming treatments for each plant species at *p* < 0.05 and *p* < 0.01, respectively, whereas no * or ** indicates non-significant difference. The lowercase letters indicate significant differences among *Medicago sativa*, *Elymus nutans* and *Hordeum vulgare* under control treatment. The uppercase letters indicate significant differences among *Medicago sativa*, *Elymus nutans* and *Hordeum vulgare* under warming treatment. No letters indicates non-significant differences among *Medicago sativa*, *Elymus nutans* and *Hordeum vulgare*.

**Figure 8:**
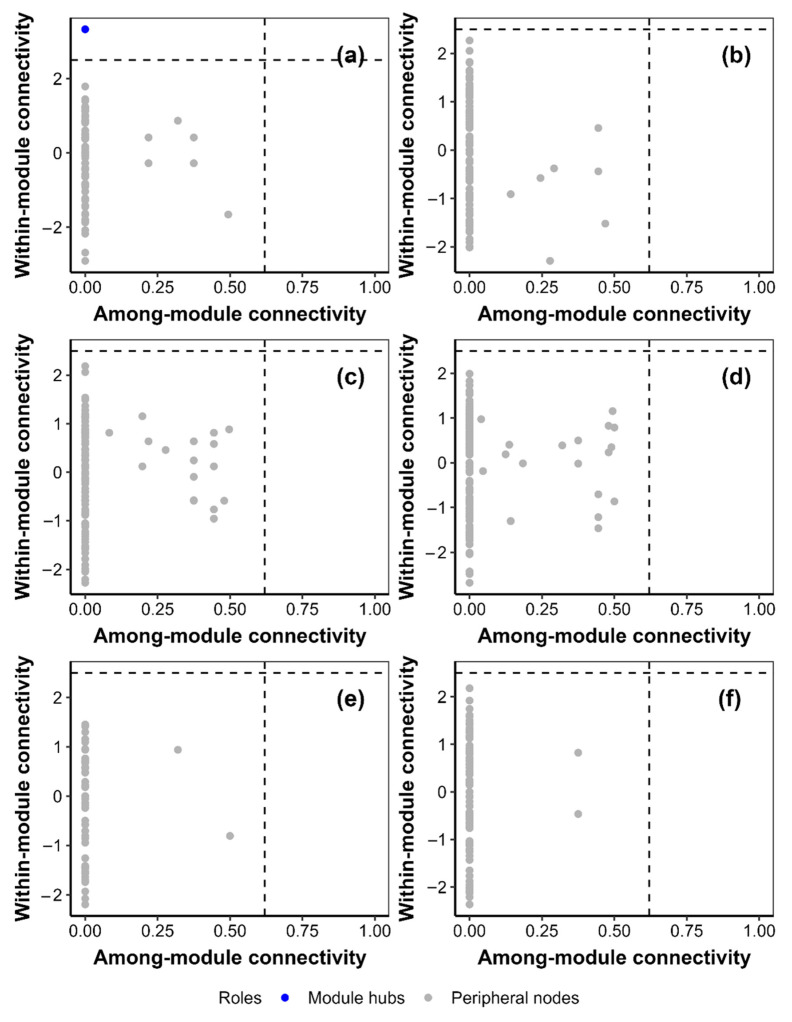
The roles of the vertex in the cooccurrence network of soil fungi community for (**a**,**b**) *Medicago sativa*, (**c**,**d**) *Elymus nutans* and (**e**,**f**) *Hordeum vulgare* within the (**a**,**c**,**e**) control and (**b**,**d**,**f**) warming plots at 0–10 cm.

**Figure 9:**
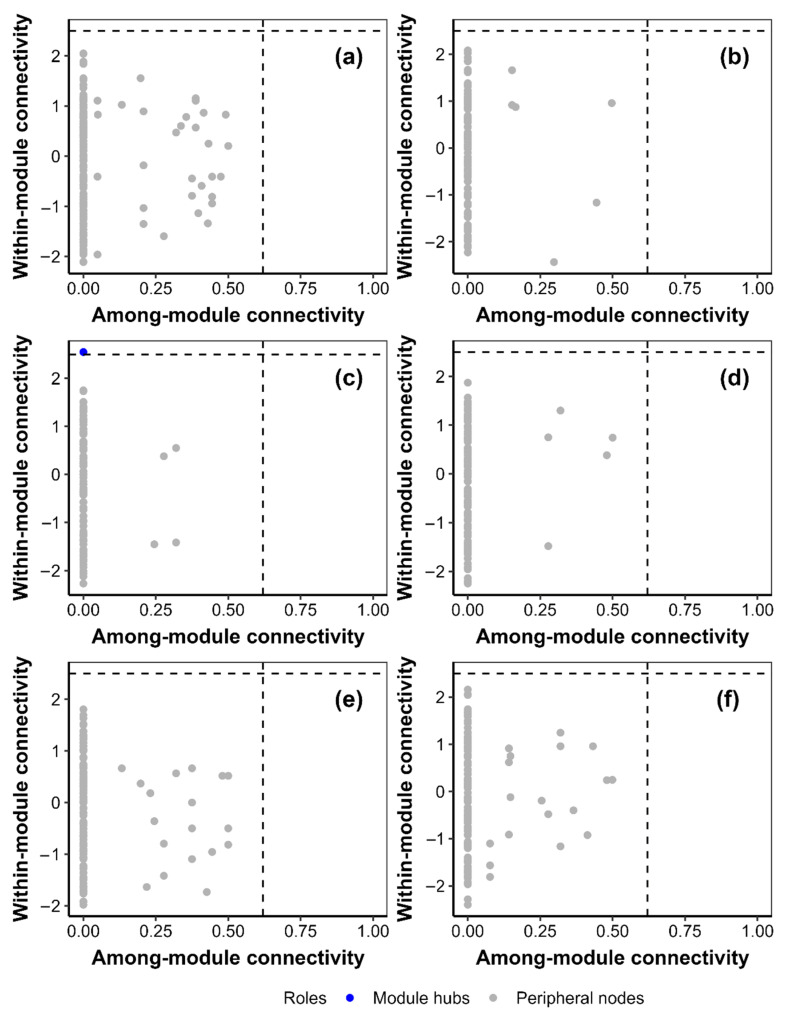
The roles of the vertex in the cooccurrence network of soil fungi community for (**a**,**b**) *Medicago sativa*, (**c**,**d**) *Elymus nutans* and (**e**,**f**) *Hordeum vulgare* within the (**a**,**c**,**e**) control and (**b**,**d**,**f**) warming plots at 10–20 cm.

Warming increased heterogeneity by 17.81% in the *Hordeum vulgare* agroecosystem at 0–10 cm and centralization by 77.51% in the *Hordeum vulgare* agroecosystem at 10–20 cm (Figure 7). Warming decreased average degree, density, heterogeneity and centralization by 51.58%, 53.44%, 26.32% and 60.40% in the *Medicago sativa* agroecosystem at 10–20 cm, respectively (Figure 7). Warming decreased the number of module hubs in the *Medicago sativa* agroecosystem at 0–10 cm and in the *Elymus nutans* agroecosystem at 10–20 cm (Figure 8 and Figure 9).

The topological parameters of the three kinds of agroecosystem are different (Figure 7). For example, edge number in the *Medicago sativa* and *Hordeum vulgare* agroecosystems was 55.87% and 60.56% lower than that of the *Elymus nutans* agroecosystem at 0–10 cm under warming conditions, respectively (Figure 7).

## 4. Discussion

This study implied that symbiotic rather than competition relationships among soil fungi species were dominant in the three agroecosystems. This phenomenon implied that there were more close cooperative rather than competition relationships among soil fungi species in the three alpine agroecosystems [11]. Such more cooperative relationships rather than competition relationships among soil fungi species may be a way for soil fungi to adapt to harsh environmental conditions in the three alpine agroecosystems.

This study found that soil fungi community varied among the three agroecosystems and each one of the three agroecosystems had its own unique species, which was in line with some previous studies [12] and may be due to their varied interactions between plant and soil fungi community [13,14]. This phenomenon implied that soil fungi diversity may increase with the increase of plant diversity, and mixed sowing may increase soil fungi diversity compared with unicast.

This study found that warming may decrease key vertices (i.e., module hubs) for soil fungi species cooccurrence network (Figure 8 and Figure 9). This phenomenon implied that warming resulted in the decrease in the complexity and stability of soil fungi cooccurrence network because the module hubs can be important indicators of the network complexity and stability of soil fungi community [11,15]. Similarly, this study also found that warming decreased average degree and density of *Medicago sativa* at 10–20 cm. This phenomenon implied that warming may decrease complex and interaction degree among species for soil fungi species cooccurrence network of the *Medicago sativa* agricultural ecosystem because the complexity of species cooccurrence is believed to be correlated with average degree [16,17]. Moreover, warming may also dampen the close relationships among species in the *Medicago sativa* agricultural ecosystem because the closeness among species is related to density [17].

However, warming did not alter complex and closeness among species of soil fungi community in the *Elymus nutans* and *Hordeum vulgare* agricultural ecosystems because warming did not alter average degree and density. Moreover, warming did not alter the vertex and edge numbers of soil fungi species cooccurrence network (Figure 7). This phenomenon implied that warming did not alter species richness and total interaction of soil fungi species cooccurrence network because the vertex and edge numbers can reflect species richness and interaction among species of soil fungi species cooccurrence network [11,18]. Third, warming also did not affect the clustering coefficient of soil fungi species cooccurrence network (Figure 7). This phenomenon implied that warming may not alter response speed of soil fungal community to external disturbance because the clustering coefficient can reflect response speed of soil fungi community to external disturbance [19,20]. Fourth, warming may not alter the efficiency of material circulation, energy flow and information transfer among soil fungi species [16,19] because warming did not alter average path length (Figure 7).

This study found that warming had negligible effects on soil fungi α-diversity and community composition in the *Hordeum vulgare* agroecosystem. This phenomenon was in line with a recent study, which showed that three-year growing-season warming had small and even negligible effects on soil fungi community in a *Hordeum vulgare* agroecosystem of Tibet [4]. This previous study ascribed such a phenomenon to short-term warming, only growing season rather than year-round warming and soil tillage before sowing [4]. However, year-round warming was used in this study. Moreover, significant positive effects of the short-term (<2 years) warming on soil fungi α-diversity were found in the *Elymus nutans* and especially *Medicago sativa* agroecosystems, and there were significant effects of the short-term (<2 years) warming on the soil fungi community composition in the *Medicago sativa* agroecosystem. Therefore, crop types can have stronger effects on the soil fungi community than warming duration, at least in the Tibet alpine agroecosystems. In addition to this, the effect of soil tillage on soil fungi community may also be important in alpine agroecosystems.

Crop types may be a very important indicator to regulate the response of soil fungi community to climate warming in agroecosystems, which can partly explain the inconsistent findings observed by previous studies in agroecosystems [21,22,23]. This phenomenon may be due to at least one of the following mechanisms. Firstly, biochar addition and soil tillage both can generally alter soil fungi community [24,25,26]. When *Hordeum vulgare* was harvested each year, above-ground biomass of *Hordeum vulgare* was completely removed from the experimental quadrats, but those were not in the other two agroecosystems. Moreover, soils were ploughed before highland barely sowing every year, while the other two agroecosystems did not. Compared with the other two agroecosystems, the soil fungi community in the *Hordeum vulgare* agroecosystem was more disturbed by human activities. Various human activities can affect soil fungi community [4]. Secondly, warming-induced changes in the species cooccurrence network varied among the three agroecosystems (Figure 7). For example, warming altered the proportion of symbiotic and competitive relationships within the soil fungi species cooccurrence network, and such changes were diverse among the three alpine agroecosystems. Thirdly, both selection and dispersal are two important ecological processes of soil fungi community assembly [27,28,29,30]. The effects of warming on ecological processes of community assembly varied among the three agroecosystems (Figure 6). Fourthly, warming can induce changes in the quantitative and qualitative composition of root exudates [31], which may in turn result in changes in soil fungi community. The amount and composition of root exudates may vary from plant to plant [13,14].

This study found that warming may homogenize or heterogenize differences of the soil fungi community among the three agroecosystems. This phenomenon can be related to the fact that there were different effects of warming on the soil fungi community among the three agroecosystems and may be due to at least one of the following mechanisms. Firstly, warming altered the differences in ecological processes of soil fungi community assembly among the three agroecosystems (Figure 6). Secondly, warming altered the differences in the species cooccurrence network of the soil fungi community among the three agroecosystems (Figure 7).

## 5. Conclusions

In summary, this was the first study to compare responses of the soil fungi community at both 0–10 cm and 10–20 cm to climate warming among *Medicago sativa*, *Elymus nutans* and *Hordeum vulgare* of Tibet. The responses of soil fungi community to warming differed between leguminous and gramineous agroecosystems and between perennial gramineous and annual gramineous agroecosystems. The initial soil fungi community varied among the three agroecosystems, and the differences of soil fungi α-diversity among the three agroecosystems at 10–20 cm were greater than those at 0–10 cm. Warming can alter the differences of soil fungi community among the three agroecosystems by increasing the differences of soil fungi α-diversity and functional composition, etc. Different depth of soil fungi community may have variable responses to climate warming. Climate warming may cause detrimental effects to the growth of perennial *Medicago sativa* by increasing the abundance of soil pathogenic fungi and decreasing the abundance of soil symbiotic and saprophytic fungi at 10–20 cm. 

## Data Availability

Request from the corresponding author.

## Supplementary Materials

The following supporting information can be downloaded at: https://www.mdpi.com/article/10.3390/jof9090885/s1, Figure S1 Venn plot for soil fungi species at 0–10 cm. AIC10: *Medicago sativa* under the control conditions; AIW10: *Medicago sativa* under the warming conditions; EDC10: *Elymus nutans* under the control conditions; EDW10: *Elymus nutans* under the warming conditions; HBC10: *Hordeum vulgare* under the control conditions; HBW10: *Hordeum vulgare* under the warming conditions. Figure S2 Venn plot for soil fungi species at 10–20 cm. AIC20: *Medicago sativa* under the control conditions; AIW20: *Medicago sativa* under the warming conditions; EDC20: *Elymus nutans* under the control conditions; EDW20: *Elymus nutans* under the warming conditions; HBC20: *Hordeum vulgare* under the control conditions; HBW20: *Hordeum vulgare* under the warming conditions. Figure S3 LEFSe analysis for soil fungi community at 0–10 cm. AIC: *Medicago sativa* under the control conditions; AIW: *Medicago sativa* under the warming conditions; EDC: *Elymus nutans* under the control conditions; EDW: *Elymus nutans* under the warming conditions; HBC: *Hordeum vulgare* under the control conditions; HBW: *Hordeum vulgare* under the warming conditions. Figure S4 LEFSe analysis for soil fungi community at 10–20 cm. AIC: *Medicago sativa* under the control conditions; AIW: *Medicago sativa* under the warming conditions; EDC: *Elymus nutans* under the control conditions; EDW: *Elymus nutans* under the warming conditions; HBC: *Hordeum vulgare* under the control conditions; HBW: *Hordeum vulgare* under the warming conditions. Figure S5 Cooccurrence network of soil fungi community of *Medicago sativa* (a, d), *Elymus nutans* (b, e) and *Hordeum vulgare* (c, f) under the control (a, b, c) and warming (d, e, f) conditions at 0–10 cm. Figure S6 Cooccurrence network of soil fungi community of *Medicago sativa* (a, d), *Elymus nutans* (b, e) and *Hordeum vulgare* (c, f) under the control (a, b, c) and warming (d, e, f) conditions at 0–10 cm. Figure S7 Venn plots for the vertex (a) and edge (b) of soil fungi cooccurrence network at 0–10 cm. AIC: *Medicago sativa* under the control conditions; AIW: *Medicago sativa* under the warming conditions; EDC: *Elymus nutans* under the control conditions; EDW: *Elymus nutans* under the warming conditions; HBC: *Hordeum vulgare* under the control conditions; HBW: *Hordeum vulgare* under the warming conditions. Figure S8 Venn plots for the vertex (a) and edge (b) of soil fungi cooccurrence network at 10–20 cm. AIC: *Medicago sativa* under the control conditions; AIW: *Medicago sativa* under the warming conditions; EDC: *Elymus nutans* under the control conditions; EDW: *Elymus nutans* under the warming conditions; HBC: *Hordeum vulgare* under the control conditions; HBW: *Hordeum vulgare* under the warming conditions. Table S1 Two-way analysis of variance for soil fungi abundance. Table S2 Two-way analysis of variance for soil fungi α-diversity. Table S3 Two-way adonis for soil fungi community composition. Table S4 Adonis between the control and warming conditions for soil fungi community composition. Table S5 Adonis among *Medicago sativa*, *Elymus nutans* and *Hordeum vulgare* for soil fungi community composition. Table S6 Two-way analysis of variance for ecological function of soil fungi community. Table S7 Two-way analysis of variance for ecological process of soil fungi community. Table S8 Two-way analysis of variance for cooccurrence network topology parameters of soil fungi community.
