# Supplementary material for: Effect of Experiment Warming on Soil Fungi Community of Medicago sativa, Elymus nutans and Hordeum vulgare in Tibet
Source: J Fungi (Basel). 2023 Aug 29;9(9):885. doi: 10.3390/jof9090885 (PMC10532768; doi:10.3390/jof9090885)
Supplement: Supplementary file 1 [file jof-09-00885-s001.zip › jof-2543938-supplementary.pdf]

Effect of experiment warming on soil fungi community of *Medicago sativa*, *Elymus nutans* and *Hordeum vulgare* in Tibet

Table S1 Two-way analysis of variance for soil fungi abundance.

Soil layers	Warming(W)		Agroecosystem (AGR)		W×AGR	
	F	*p*	*F*	*p*	*F*	*p*
0–10 cm	1.09	0.307	**11.14**	**<0.001**	0.12	0.890
10–20 cm	0.20	0.657	0.42	0.661	2.70	0.087

Table S2 Two-way analysis of variance for soil fungi α-diversity.

Soil layers	Diversity		Warming(W)		Agroecosystem (AGR)		W×AGR	
			F	*p*	*F*	*p*	*F*	*p*
0–10 cm	Taxonomy α-diversity	OTU	0.70	0.411	2.53	0.100	0.13	0.880
		$Chao1_t$	0.69	0.415	0.93	0.407	0.13	0.881
		ACE_t	0.81	0.378	0.93	0.410	0.06	0.941
		$Shannon_t$	0.07	0.793	**4.86**	**0.017**	0.19	0.830
		$Simpson_t$	0.06	0.809	**4.89**	**0.017**	0.65	0.533
	Phylogeny α-diversity	PD	0.67	0.423	2.28	0.124	0.13	0.876
		MPD	0.05	0.820	**4.73**	**0.019**	0.37	0.692
		MNTD	0.00	0.976	3.09	0.064	0.10	0.907
	Function α-diversity	Function number	0.06	0.816	1.44	0.257	0.54	0.589
		$Chao1_f$	0.01	0.924	1.02	0.375	1.05	0.365
		ACE_f	0.20	0.655	0.31	0.738	1.36	0.275
		$Shannon_f$	0.02	0.879	1.56	0.232	0.16	0.855
		$Simpson_f$	0.00	0.984	1.62	0.219	0.21	0.815

10–20 cm	Taxonomy α-diversity	OTU	0.90	0.353	0.39	0.680	1.51	0.242
		$Chao1_t$	0.11	0.739	0.49	0.620	0.34	0.718
		ACE_t	0.13	0.717	0.36	0.699	0.82	0.453
		$Shannon_t$	1.52	0.229	1.39	0.268	1.07	0.360
		$Simpson_t$	0.95	0.340	1.38	0.272	0.63	0.543
	Phylogeny α-diversity	PD	1.23	0.279	0.30	0.746	1.36	0.275
		MPD	1.00	0.328	3.10	0.064	1.49	0.246
		MNTD	0.09	0.768	0.98	0.391	1.16	0.332
	Function α-diversity	Function number	2.94	0.099	0.30	0.746	1.22	0.313
		$Chao1_f$	1.98	0.173	0.35	0.707	0.60	0.559
		ACE_f	**4.86**	**0.037**	1.92	0.168	0.50	0.614
		$Shannon_f$	**4.38**	**0.047**	2.09	0.145	2.98	0.070
		$Simpson_f$	**4.49**	**0.045**	2.12	0.142	2.62	0.093

Table S3 Two-way adonis for soil fungi community composition.

	Depth	Warming(W)			Agroecosystem (AGR)			W×AGR		
		R^2	F	p	R^2	F	p	R^2	F	p
Taxonomy composition	0–10 cm	0.02	0.72	0.824	0.09	1.29	0.118	0.06	0.89	0.652
	10–20 cm	0.04	1.23	0.225	0.07	0.99	0.464	0.07	1.04	0.408
Phylogeny composition	0–10 cm	0.04	1.24	0.376	0.05	0.68	0.672	0.08	1.15	0.390
	10–20 cm	0.09	2.18	0.282	-0.07	-0.92	0.945	0.01	0.15	0.744
Function composition	0–10 cm	0.01	0.41	0.776	0.14	2.13	0.081	0.04	0.64	0.658
	10–20 cm	**0.08***	**2.67**	**0.043**	0.07	1.07	0.352	0.09	1.43	0.186

Table S4 Adonis between the control and warming conditions for soil fungi community composition.

	Depth	Medicago sativa			Elymus nutans			Hordeum vulgare		
		R^2	F	p	R^2	F	p	R^2	F	p
Taxonomy composition	0–10 cm	0.83	0.09	0.928	0.92	0.10	0.632	0.90	0.10	0.740
	10–20 cm	**1.36***	**0.14**	**0.017**	0.94	0.11	0.555	0.96	0.11	0.505
Phylogeny composition	0–10 cm	1.04	0.11	0.479	1.39	0.15	0.400	0.71	0.08	0.661
	10–20 cm	0.88	0.10	0.625	0.79	0.09	0.428	0.74	0.08	0.567
Function composition	0–10 cm	0.61	0.07	0.833	0.35	0.04	0.838	1.11	0.12	0.352
	10–20 cm	**4.12***	**0.34**	**0.023**	0.95	0.11	0.508	0.41	0.05	0.768

Table S5 Adonis among Medicago sativa, Elymus nutans and Hordeum vulgare for soil fungi community composition.

	Depth	Control			Warming		
		R^2	F	p	R^2	F	p
Taxonomy composition	0-10 cm	1.15	0.16	0.117	1.08	0.15	0.239

	10-20 cm	0.91	0.13	0.709	1.16	0.16	0.114
Phylogeny composition	0-10 cm	1.15	0.16	0.348	0.82	0.12	0.641
	10-20 cm	0.37	0.06	0.835	-0.81	-0.16	0.922
Function composition	0-10 cm	1.74+	0.22	0.095	0.86	0.13	0.535
	10-20 cm	0.91	0.13	0.483	**2.05***	**0.26**	**0.033**

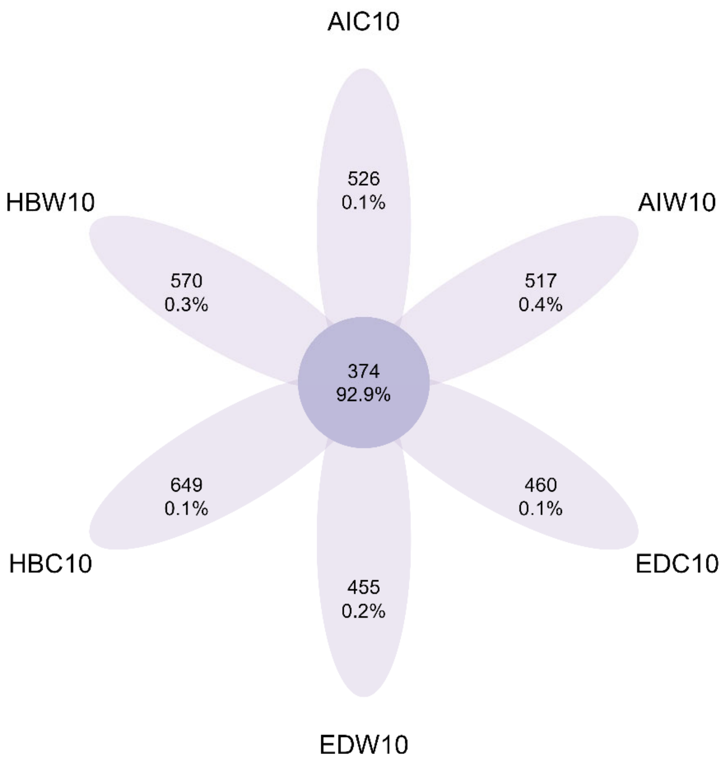

Figure S1 Venn plot for soil fungi species at 0–10 cm. AIC10: Medicago sativa under the control conditions; AIW10: Medicago sativa under the warming conditions; EDC10: Elymus nutans under the control conditions; EDW10: Elymus nutans under the warming conditions; HBC10: Hordeum vulgare under the control conditions; HBW10: Hordeum vulgare under the warming conditions.

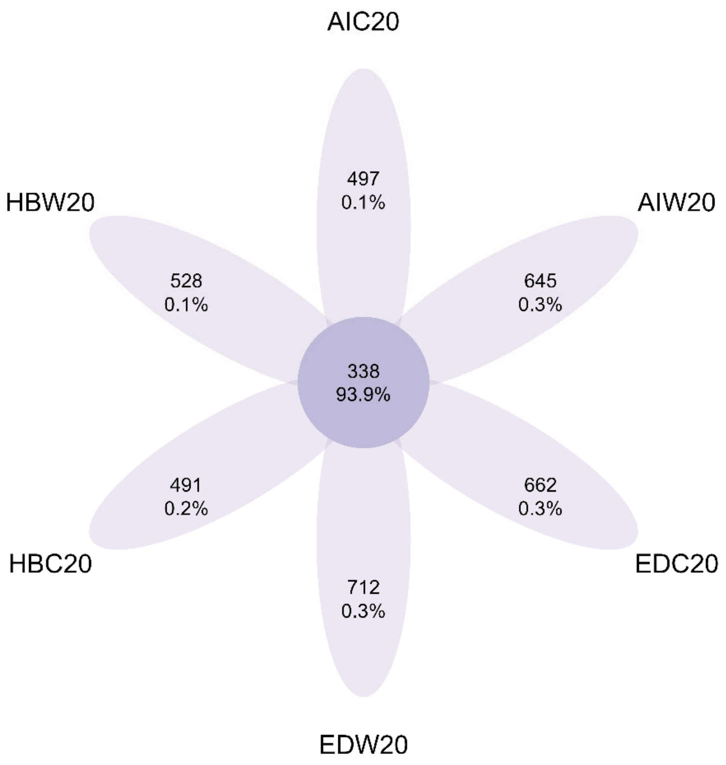

Figure S2 Venn plot for soil fungi species at 10–20 cm. AIC20: Medicago sativa under the control conditions; AIW20: Medicago sativa under the warming conditions; EDC20: Elymus nutans under the control conditions; EDW20: Elymus nutans under the warming conditions; HBC20: Hordeum vulgare under the control conditions; HBW20: Hordeum vulgare under the warming conditions.

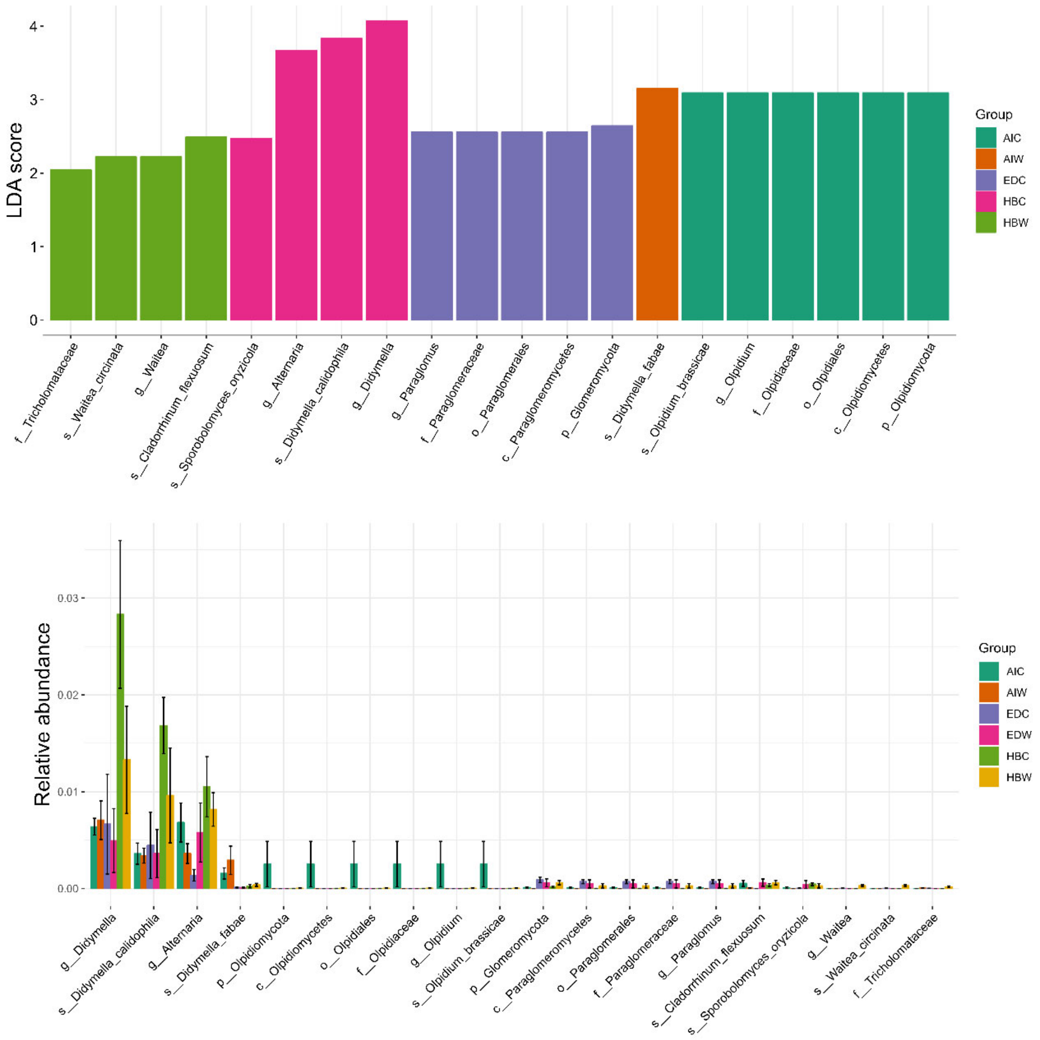

Figure S3 LEFSe analysis for soil fungi community at 0–10 cm. AIC: Medicago sativa under the control conditions; AIW: Medicago sativa under the warming conditions; EDC: Elymus nutans under the control conditions; EDW: Elymus nutans under the warming conditions; HBC: Hordeum vulgare under the control conditions; HBW: Hordeum vulgare under the warming conditions.

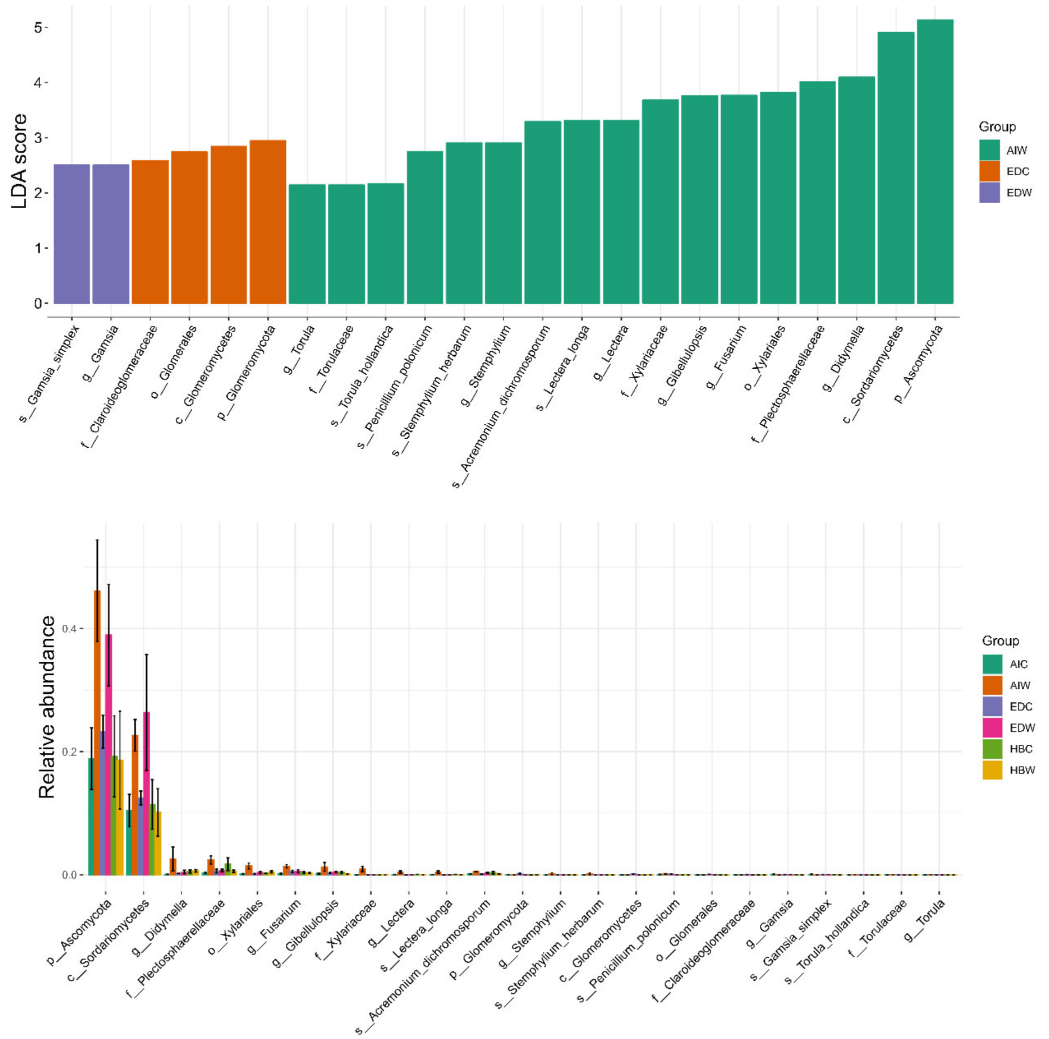

Figure S4 LEFSe analysis for soil fungi community at 10–20 cm. AIC: Medicago sativa under the control conditions; AIW: Medicago sativa under the warming conditions; EDC: Elymus nutans under the control conditions; EDW: Elymus nutans under the warming conditions; HBC: Hordeum vulgare under the control conditions; HBW: Hordeum vulgare under the warming conditions.

Table S6 Two-way analysis of variance for ecological function of soil fungi community.

Depth	Fungi function	Warming(W)		Agroecosystem (AGR)		W×AGR	
		F	*p*	*F*	*p*	*F*	*p*
0–10 cm	Symbiotroph	0.04	0.834	1.63	0.217	0.98	0.390
	Pathotroph	0.45	0.508	2.35	0.117	0.34	0.713
	Saprotroph	0.03	0.857	2.19	0.134	0.37	0.695
	Pathotroph-Symbiotroph	**4.28**	**0.050**	0.02	0.981	0.00	0.997
	Saprotroph-Symbiotroph	0.18	0.676	2.98	0.070	0.42	0.664
	Pathotroph-Saprotroph	0.79	0.383	1.72	0.201	0.10	0.904
	Pathotroph-Saprotroph-Symbiotroph	1.43	0.244	2.77	0.083	0.16	0.856
10–20 cm	Symbiotroph	**5.04**	**0.034**	**6.17**	**0.007**	**6.17**	**0.007**
	Pathotroph	2.30	0.142	1.24	0.306	1.13	0.339
	Saprotroph	0.95	0.340	2.22	0.130	1.66	0.212
	Pathotroph-Symbiotroph	2.61	0.119	**3.77**	**0.038**	1.96	0.163
	Saprotroph-Symbiotroph	**4.35**	**0.048**	1.32	0.285	1.81	0.185
	Pathotroph-Saprotroph	3.68	0.067	2.39	0.113	1.40	0.267
	Pathotroph-Saprotroph-Symbiotroph	2.08	0.162	1.77	0.191	**4.32**	**0.025**

Table S7 Two-way analysis of variance for ecological process of soil fungi community.

Depth	Ecological process	Warming(W)		Agroecosystem (AGR)		W×AGR	
		F	*p*	*F*	*p*	*F*	*p*
0–10 cm	Heterogeneous selection	1.36	0.249	2.73	0.074	1.30	0.280
	Homogeneous selection	**4.18**	**0.046**	0.25	0.780	2.71	0.076
	Dispersal limitation	0.71	0.404	**7.41**	**0.001**	0.21	0.815
	Homogenizing dispersal	0.53	0.471	0.70	0.502	1.90	0.159
	Drift & others	**4.42**	**0.040**	**5.75**	**0.005**	1.38	0.261
10–20 cm	Heterogeneous selection	0.61	0.439	2.42	0.099	**6.05**	**0.004**
	Homogeneous selection	1.37	0.247	**4.36**	**0.018**	**4.97**	**0.010**
	Dispersal limitation	2.96	0.091	0.09	0.912	**4.42**	**0.017**
	Homogenizing dispersal	2.14	0.149	2.64	0.081	1.19	0.311
	Drift & others	1.06	0.309	0.12	0.890	2.69	0.077

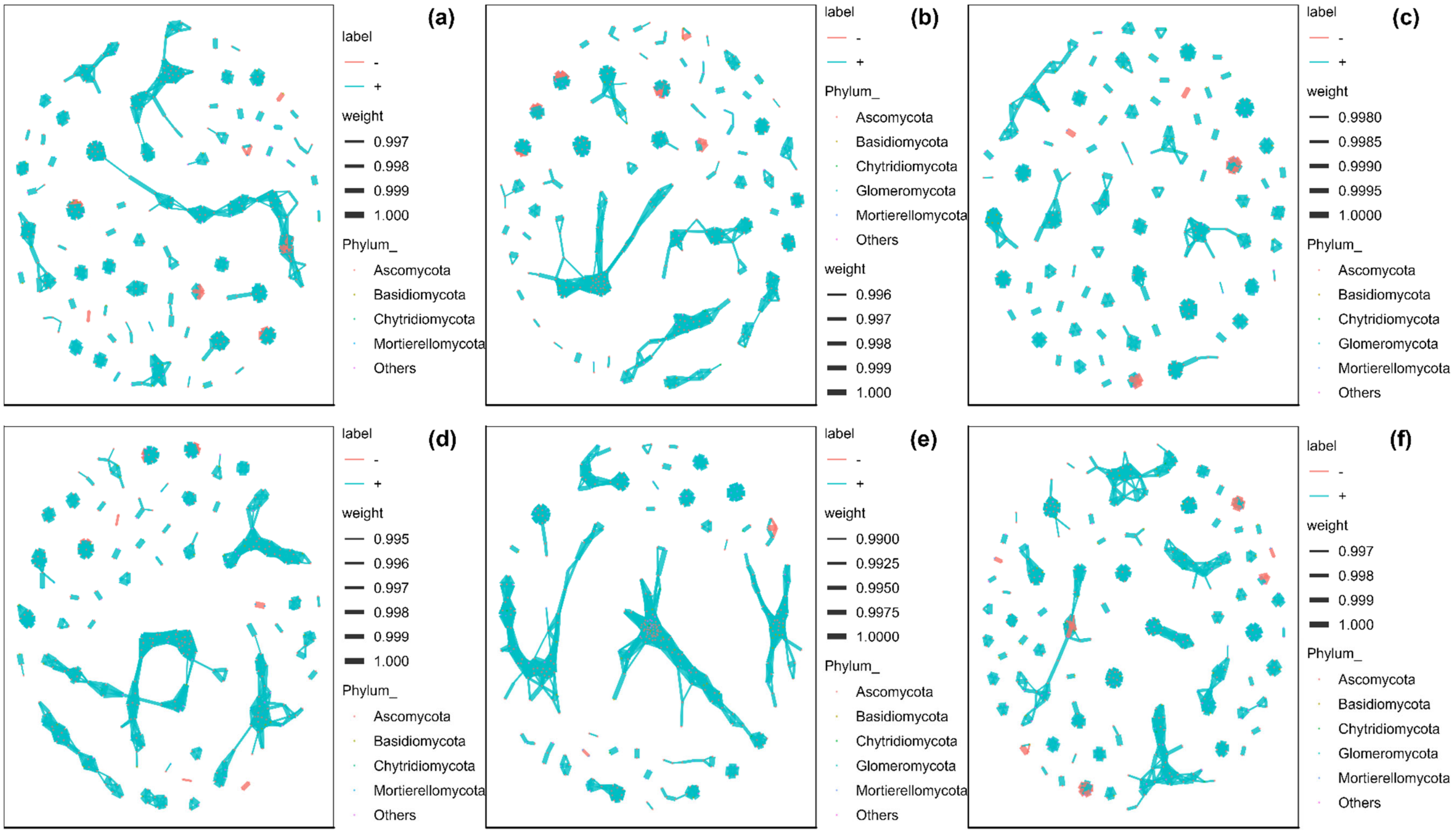

Figure S5 Cooccurrence network of soil fungi community of Medicago sativa (a, d), Elymus nutans (b, e) and Hordeum vulgare (c, f) under the control (a, b, c) and warming (d, e, f) conditions at 0–10 cm.

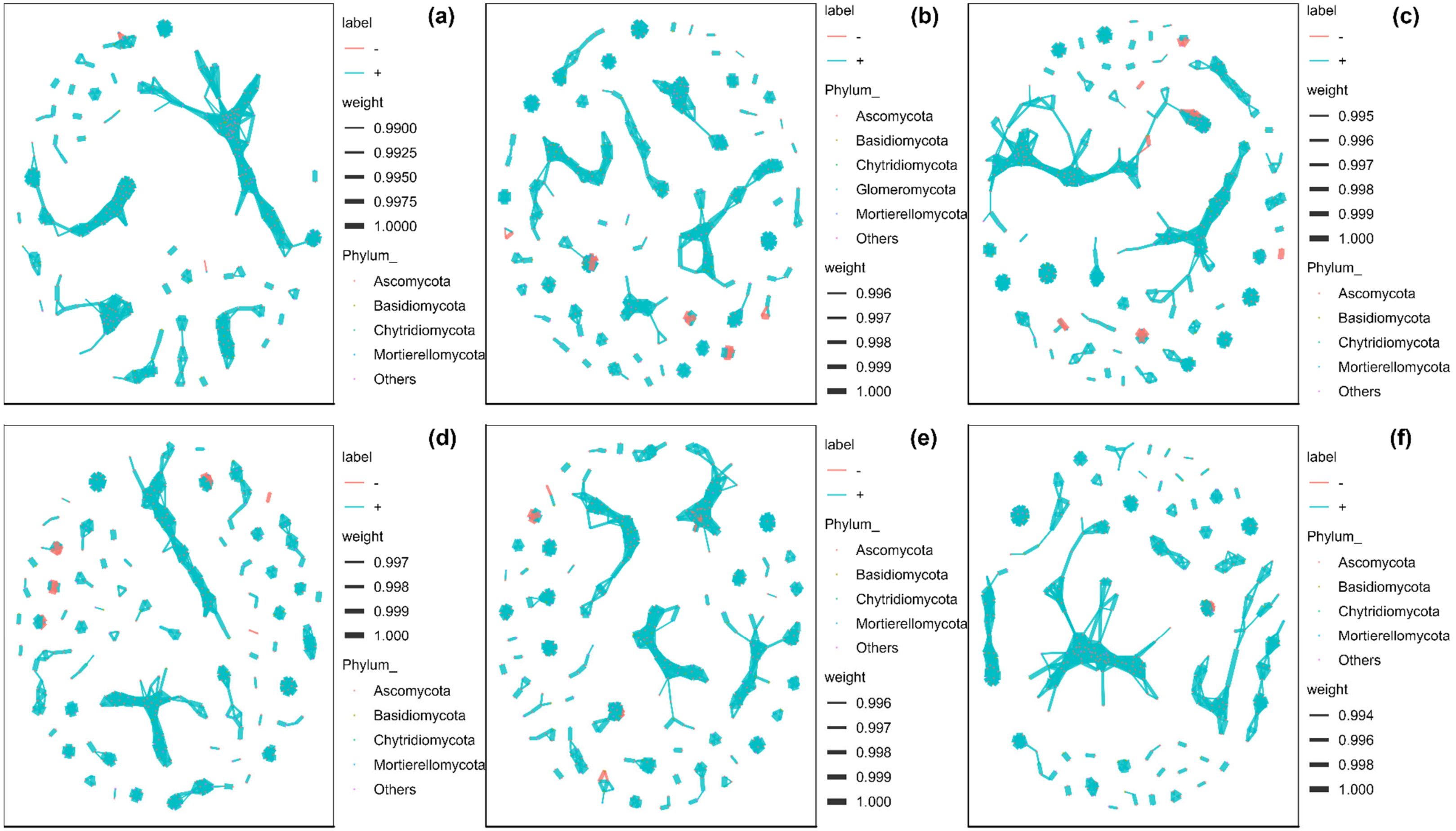

Figure S6 Cooccurrence network of soil fungi community of Medicago sativa (a, d), Elymus nutans (b, e) and Hordeum vulgare (c, f) under the control (a, b, c) and warming (d, e, f) conditions at 10–20 cm.

Table S8 Two-way analysis of variance for cooccurrence network topology parameters of soil fungi community.

Depth	Ecological process	Warming(W)		Agroecosystem (AGR)		W×AGR	
		F	*p*	*F*	*p*	*F*	*p*
0–10 cm	Vertex	0.69	0.413	0.18	0.837	0.51	0.608
	Edge	**6.56**	**0.017**	**4.65**	**0.020**	1.97	0.162
	Average degree	**6.76**	**0.016**	**5.05**	**0.015**	2.35	0.117
	Average path length	1.26	0.273	2.87	0.077	0.20	0.819
	Network diameter	1.55	0.226	2.42	0.110	0.06	0.945
	Clustering coefficient	3.63	0.069	2.12	0.142	0.99	0.388
	Density	**4.37**	**0.047**	2.94	0.072	1.93	0.167
	Heterogeneity	**6.47**	**0.018**	**5.89**	**0.008**	0.16	0.853
	Centralization	**9.33**	**0.005**	**8.02**	**0.002**	1.65	0.213
10–20 cm	Vertex	0.01	0.928	0.54	0.588	0.27	0.765
	Edge	0.51	0.484	1.19	0.321	2.00	0.157
	Average degree	1.42	0.246	2.11	0.144	**5.34**	**0.012**
	Average path length	1.41	0.246	1.33	0.284	0.08	0.919
	Network diameter	0.91	0.349	0.77	0.473	0.22	0.806
	Clustering coefficient	**7.10**	**0.014**	0.01	0.991	0.14	0.874
	Density	4.08	0.055	**4.56**	**0.021**	**9.75**	**<0.001**
	Heterogeneity	0.42	0.522	2.57	0.097	**8.27**	**0.002**
	Centralization	2.77	0.109	**4.64**	**0.020**	**11.57**	**<0.001**

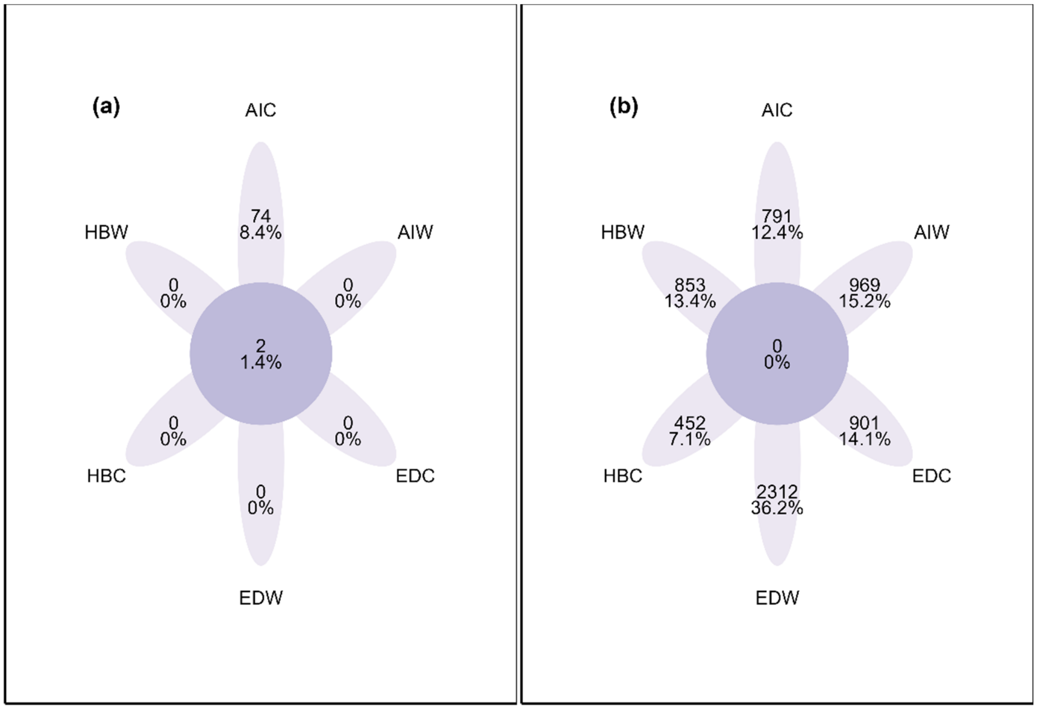

Figure S7 Venn plots for the vertex (a) and edge (b) of soil fungi cooccurrence network at 0–10 cm. AIC: Medicago sativa under the control conditions; AIW: Medicago sativa under the warming conditions; EDC: Elymus nutans under the control conditions; EDW: Elymus nutans under the warming conditions; HBC: Hordeum vulgare under the control conditions; HBW: Hordeum vulgare under the warming conditions.

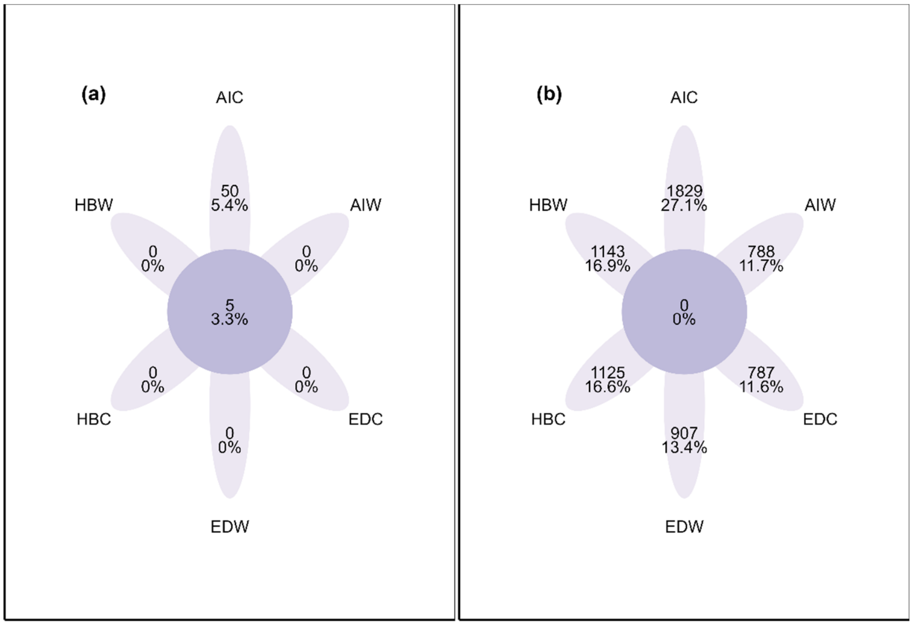

Figure S8 Venn plots for the vertex (a) and edge (b) of soil fungi cooccurrence network at 10–20 cm. AIC: Medicago sativa under the control conditions; AIW: Medicago sativa under the warming conditions; EDC: Elymus nutans under the control conditions; EDW: Elymus nutans under the warming conditions; HBC: Hordeum vulgare under the control conditions; HBW: Hordeum vulgare under the warming conditions.
